# Astaxanthin vs placebo on arterial stiffness, oxidative stress and inflammation in renal transplant patients (Xanthin): a randomised controlled trial

**DOI:** 10.1186/1471-2369-9-17

**Published:** 2008-12-18

**Authors:** Robert G Fassett, Helen Healy, Ritza Driver, Iain K Robertson, Dominic P Geraghty, James E Sharman, Jeff S Coombes

**Affiliations:** 1 Renal Research, Royal Brisbane and Women's Hospital, Herston, Brisbane, Queensland, Australia; 2 Renal Research Tasmania, Launceston General Hospital, Launceston, Tasmania, Australia; 3 School of Human Life Sciences, University of Tasmania, Launceston, Tasmania, Australia; 4 School of Human Movement Studies, University of Queensland, St. Lucia, Queensland, Australia; 5 School of Medicine, University of Queensland, St. Lucia, Queensland, Australia

## Abstract

**Background:**

There is evidence that renal transplant recipients have accelerated atherosclerosis manifest by increased cardiovascular morbidity and mortality. The high incidence of atherosclerosis is, in part, related to increased arterial stiffness, vascular dysfunction, elevated oxidative stress and inflammation associated with immunosuppressive therapy. The dietary supplement astaxanthin has shown promise as an antioxidant and anti-inflammatory therapeutic agent in cardiovascular disease. The aim of this trial is to investigate the effects of astaxanthin supplementation on arterial stiffness, oxidative stress and inflammation in renal transplant patients.

**Method and Design:**

This is a randomised, placebo controlled clinical trial. A total of 66 renal transplant recipients will be enrolled and allocated to receive either 12 mg/day of astaxanthin or an identical placebo for one-year. Patients will be stratified into four groups according to the type of immunosuppressant therapy they receive: 1) cyclosporine, 2) sirolimus, 3) tacrolimus or 4) prednisolone+/-azathioprine, mycophenolate mofetil or mycophenolate sodium. Primary outcome measures will be changes in 1) arterial stiffness measured by aortic pulse wave velocity (PWV), 2) oxidative stress assessed by plasma isoprostanes and 3) inflammation by plasma pentraxin 3. Secondary outcomes will include changes in vascular function assessed using the brachial artery reactivity (BAR) technique, carotid artery intimal medial thickness (CIMT), augmentation index (AIx), left ventricular afterload and additional measures of oxidative stress and inflammation. Patients will undergo these measures at baseline, six and 12 months.

**Discussion:**

The results of this study will help determine the efficacy of astaxanthin on vascular structure, oxidative stress and inflammation in renal transplant patients. This may lead to a larger intervention trial assessing cardiovascular morbidity and mortality.

**Trial Registration:**

ACTRN12608000159358

## Background

Vascular disease is the leading cause of morbidity and mortality in renal transplant recipients [1]. Measures of arterial stiffness such as aortic pulse wave velocity (PWV) predict morbidity and mortality in patients with kidney disease [2]. In addition, numerous studies have reported elevated levels of oxidative stress and inflammation in this population [3-7] and this has been associated with arterial stiffness [8]. Our research group has shown that cyclosporine, a commonly used immunosuppressant taken by renal transplant recipients, increases markers of oxidative stress [9,10] and decreases vascular function [11]. In addition, antioxidant supplementation reduced oxidative stress and protected against the cyclosporine-induced vascular dysfunction [11].

Nutritional antioxidants have the ability to decrease lipid and protein oxidation, potentially protecting against atherosclerosis and arterial stiffening [12]. Although data from epidemiological [13-16], animal [17] and *in vitro *[18] studies has supported the notion that an increased antioxidant intake will decrease the risk of atherosclerosis and vascular disease, large randomised controlled trials have not supported this postulate [19,20]. Thus, more research is needed, especially in high-risk groups such as renal transplant recipients. Importantly, new antioxidants with different biological actions need to be investigated.

Astaxanthin is a carotenoid, belonging to the same family as beta-carotene. Compared with other carotenoids it contains two additional oxygenated groups on each ring structure, resulting in enhanced antioxidant properties [21]. The compound occurs naturally in a wide variety of living organisms including microalgae, fungi, complex plants and crustaceans. It is reddish-coloured and gives salmon, shrimp and lobster their distinctive appearance. It quenches reactive oxygen and nitrogen species, single and two electron oxidants, and is a chain breaker of free radicals. The most common source of astaxanthin used in dietary supplements is from Haematococcus alga. The United States Food and Drug Administration approved astaxanthin as a nutaceutical [21]. Recently astaxanthin, which has up to 500 times the potency of vitamin E, has been suggested to play a valuable role in antioxidant protection of cells and in protection against cardiovascular disease [22].

Preclinical studies have demonstrated that astaxanthin supplementation has anti-inflammatory properties [23,24] and potential efficacy in ischemia-reperfusion [25,26], reduction of lipid peroxidation [27,28], and rethrombosis after thrombolysis [29]. Only a few studies have investigated the potential benefits of astaxanthin in human health (Table 1). It has been shown that astaxanthin reduces reflux symptoms [30], improves age-related macular degerenaration [31] and sperm parameters [32]. Another study reported astaxanthin supplementation had no effect on muscle injury following intense exercise [33]. In addition, studies conducted in humans and animals have found significant reductions in oxidative stress, dyslipidemia and inflammatory markers after oral supplementation with astaxanthin [27,34,35].

**Table 1:** Clinical studies investigating the effects astaxanthin

Study	Dosage	Duration of supplementation	Findings
Iwamoto et al. 2000 [27]	Different doses: 1.8, 3.6, 14.4, 21.6 mg/day	2 weeks	Inhibition of LDL oxidation
Mercke Odeberg et al. 2003 [49]	40 mg	Single dose	Enhanced bioavailability with lipid based formulation
Spiller et al. 2003 [36]	6 mg/day (3 × 2 mg tablets/day)	8 weeks	Safety demonstrated in humans over 8 weeks
Comhaire et al. 2005 [32]	16 mg/day	12 weeks	Improved sperm parameters
Bloomer et al. 2005 [33]	4 mg/day	12 weeks	No effect on muscle injury
Andersen et al. 2007 [35]	40 mg/day	8 weeks	No effect on H pylori density or gastric inflammation
Karppi et al. 2007 [34]	8 mg/day	12 weeks	Intestinal absorption adequate with Capsules. Decreases oxidation of fatty acids
Kupcinskas et al. 2008 [30]	16 mg or 40 mg/day	4 weeks	Greater reduction in reflux symptoms in 40 mg treated patients and more pronounced if H pylori positive
Parisi et al. 2008 [31]	4 mg/day	12 months	Improved central retinal dysfunction in age related macular degeneration when administered with other antioxidants

The studies in humans have used astaxanthin in a range of different doses, for various durations with no reported side effects (Table 1). A study conducted in 35 healthy adults specifically investigated the safety of astaxanthin supplementation [36]. The study was a randomised, double blind, placebo controlled trial in which participants were required to consume 6 mg of astaxanthin per day for eight weeks. Measures of blood pressure and blood chemistry conducted at four and eight weeks, revealed no significant differences between the treatment and placebo group and the authors concluded that 6 mg of astaxanthin per day from a Haematococcus pluvialis algal extract can be safely consumed by healthy adults.

At this stage, little is known regarding the effectiveness of antioxidant supplementation on vascular disease in renal transplant patients. A recent study from our research team showed that antioxidant supplementation had an effect on blood cyclosporine concentrations and renal function [37]. Therefore, additional studies that improve our understanding of antioxidant efficacy may lead to important clinical benefits in the prevention of atherosclerosis and vascular disease in these patients. The purpose of this study is to assess the effect of antioxidant supplementation with astaxanthin on arterial stiffness, oxidative stress and inflammation in renal transplant patients.

## Methods and design

### Study Design and Setting

The Xanthin study is a multi-centre, one-year double blind randomized placebo controlled trial in subjects with a renal transplant. The study is being conducted at the Launceston General Hospital and Burnie Satellite Renal Units in Northern Tasmania, which service a population of approximately 250,000.

### Ethical Considerations

The Tasmanian Statewide Scientific and Ethics Committees approved the Xanthin Trial. The Ethics Committees will be provided with annual reports of the trial progress and will promptly receive all adverse event reports.

### Identification of Eligible Patients

The principal investigators and the clinical trial coordinators will screen the medical records of patients prior to their attendance at the renal clinics. Eligible patients will have a copy of the patient information sheet placed in the medical record. The principal investigator will then explain the study during the clinical consultation. After the explanation the subject will be provided with a patient information sheet and informed consent form. The subject will then be asked to take this away with them and arrangements will be made to follow up via a telephone call. If the subject agrees to participate, they will sign the consent form with an independent person signing as a witness.

### Eligibility

Inclusion criteria are; age > 18 and < 85 years having undergone renal transplantation. Subjects will be excluded if they are already taking antioxidant supplementation, unable to take glyceryl trinitrate or are participating in, or propose to participate in, another clinical drug study within 30 days prior to study entry.

### Randomization

The clinical trial pharmacist at the Launceston General Hospital or Royal Brisbane and Women's, who is independent of the study team, will perform the randomization. Subjects will also be stratified according to four groups according to the type of immunosuppression used for the renal transplant. The four groups will be those taking 1) cyclosporine, 2) tacrolimus, 3) sirolimus and 4) prednisolone together with either azathioprine, mycophenolate mofetil or mycophenolate sodium. Computer generated random numbers will be placed in blocks of ten by the clinical trial pharmacist and related to a series of drug code numbers. Each drug code assignment block will refer to one of the stratification groups. Data recorded will include the drug code number, which becomes the subjects Xanthin trial identification number, the patient hospital record number, the date of allocation, and the type of immunosuppressant used. The sheet containing the blocks of drug code numbers are given to the clinical trial coordinator who thus is blinded to the allocation performed by the pharmacist. Once the immunosuppressant type is confirmed by the principal investigator the clinical trial pharmacist selects the next available drug code number from the relevant stratification block according to the type of immunosuppressant. This enables the pharmacist at the patient's location to dispense from the appropriate randomization group. The subject receives a specific purpose numbered Xanthin trial plastic container with tablets enclosed, which are indistinguishable as to whether they contain astaxanthin or placebo. The randomization code is kept sealed in an opaque envelope in the Launceston General Hospital and an identical copy is kept at an off-site location of one of the associate investigators for the duration of the study.

### Study Medication and Dosing

Cyanotech Corporation provided the astaxanthin (BioAstin) and identical placebo, which were shipped in containers from Hawaii. The astaxanthin is derived from *haematococcus pluvialis *(microalge). Tablets will be checked to ensure they will not exceed their expiry date at any stage of their prescription to subjects. They will then be placed in number coded containers so it is not possible to distinguish, other than with the code, which container has placebo or astaxanthin. Each group will take three tablets (at one time point) per day. The active treatment is 12 mg of astaxanthin per day (3 × 4 mg tablets).

### Primary Objectives and Primary Outcome Measures

The primary objectives are to assess the effects of astaxanthin on arterial stiffness, oxidative stress and inflammation. The primary outcome measures will be outlined below. The hypotheses are that astaxanthin will significantly improve the rates of change in these measures compared with controls.

#### Arterial stiffness

Carotid to femoral PWV will be derived by electrocardiography-gated sequential applanation tonometry (SPT-301 Mikro-Tip, Millar Instruments, Houston, Texas) using the foot-to-foot method (SphymoCor™ 7.01 AtCor, Sydney, Australia) [38]. Testing is performed in a temperature-controlled room and after 5–10 minutes rest with the subject in the supine position prior to the first measure. Two measures will be averaged for the estimation of aortic PWV.

#### Oxidative stress and inflammation

The primary outcome measure for oxidative stress will plasma isoprostanes measured using gas chromatography mass spectrometry [39] and for inflammation, pentraxin 3 using an ELISA assay [40].

### Secondary Objectives and Secondary Outcome Measures

Secondary objectives of the Xanthin trial are to assess the effects of astaxanthin on vascular function and additional measures of arterial stiffness, left ventricular afterload, oxidative stress and inflammation.

#### Vascular function

Brachial artery reactivity (BAR) will be measured to assess vascular function. A 12 MHz linear array transducer and ultrasound scanner (Vivid i, GE Healthcare, USA) will be used to record the dilator response of the brachial artery to increased blood flow generated during reactive hyperaemia of the downstream forearm. The measures are performed on the non-fistula arm; if one is still present. A blood pressure cuff is placed on the upper forearm. The brachial artery will be scanned in the longitudinal plane, acquiring a baseline image about 5 cm above the antecubital fossa. The transducer is left in this scanning position. The cuff is then inflated to 220 mmHg for 4 minutes. Following cuff deflation, images will be acquired at 5 and 10 seconds then at 10-second intervals up to 120 seconds. Following a 15-minute rest, this process is then repeated. This allows the artery to return to baseline state. After another 15 minutes an additional baseline image is acquired and then 300 μg of sublingual GTN will be administered. After 3 minutes images of the brachial artery are obtained as described above. All images are stored for later offline analysis. The truest diameter will evidenced by the "double line sign", i.e. crisp intima-media and media-adventitia boundaries on the near and far walls [41]. For all measures, the average of three diameter measurements made along the artery, from the trailing edge of intima-media to the leading edge of the media-adventitia, as described by Aeschlimann et al [41], will be used. BAR will be expressed as maximal dilatation (mm), as a percentage change from baseline. The area under the diameter/time curve (AUC) will also be used to determine temporal changes using analytical imaging software (Imaging Research, St. Catherine's, Canada) and confirming with trapezoidal integration in Microsoft Excel (Seattle, WA, USA) as we have done previously [42]. These will then be compared with the dilatation response caused by the administration of sublingual glyceryl trinitrate (GTN). This gives a ratio of endothelial dependant and independent vascular function.

#### Arterial Stiffness and Left Ventricular Afterload

Carotid artery intimal medial thickness (CMIT) and augmentation index (AIx) will be measured to further assess arterial stiffness. Central pulse pressure will be used to determine left ventricular afterload.

CIMT refers to the combined thickness of the intimal and medial layers of the carotid arterial wall [43]. This corresponds to the inner and outer echogenic lines seen on the B-mode ultrasound image [44]. The CMIT will be assessed using a 12 MHz linear array transducer and ultrasound scanner (Vivid i, GE Healthcare, USA). Images will be acquired from the anterior, posterior and lateral planes of the right common carotid artery. The thickness will be measured one to two centimetres proximal to the bifurcation and during cardiac diastole from the far wall only as there are technical and acoustic difficulties encountered when measuring the near wall thickness [44]. For the purpose of the analysis, the average of these three recordings will be used. CIMT assessment will be performed in the plaque-free arterial wall (an atherosclerotic plaque being defined as an echo-structure protruding into the vascular lumen and a thickness greater by at least 50% than neighbouring sites [45].

Pulse wave analysis (PWA) will be used to derive a central (ascending aortic) pressure waveform via applanation tonometry at the radial artery [38] to determine central pulse pressure and AIx as a composite measure of arterial stiffness. This method involves uses a generalised transfer function to transform the radial pressure waveform. The central waveform will be calibrated by the average of two measures of brachial blood pressure using a semi-automated device (UA-767, A&D).

#### Oxidative Stress and Inflammation

Additional secondary outcome measures for oxidative stress and antioxidant status will include plasma protein carbonyls, total antioxidant capacity, antioxidant enzyme activities, and for inflammation, C reactive protein and a cytokine panel consisting of IL-6, IL-8, IL-10 and TNF-alpha.

Additional data to be collected will include self-reported health (SF-36 questionnaire), physical activity levels (items from the Active Australia questionnaire) nutritional status (four-day diet diary), cardiovascular events, mortality, hospital admissions, supplement safety and tolerability. Based on our previous finding where antioxidant supplementation with vitamin E resulted in a reduction in cyclosporine levels [37], patients will have a blood sample taken one week after starting therapy to measure immunosuppressant levels to assess whether any adverse changes occur.

### Visit One (baseline data)

**Figure 1:**
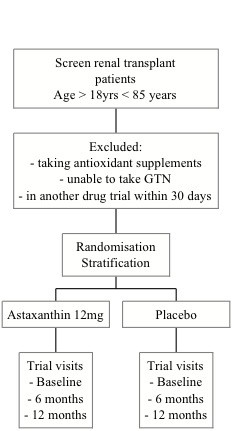
Flow chart of Xanthin trial.

The Xanthin study flow is summarized in Figure 1 and the study evaluations are outlined in Table 2. After obtaining informed consent patients will be contacted by telephone by the trial coordinator at which a date and time is agreed for subject to attend an appointment for baseline trial measures. A letter of confirmation of this appointment and a Xanthin Trial pathology request form will be posted. Patients will be asked to attend the pathology laboratory at least seven days before their first trial visit to have a fasting blood sample collected. At the first trial visit, additional data will be obtained from the medical records (medical history, medications) and measures of height, weight and blood pressure will be recorded. PWV, PWA, CIMT and BAR measures will then be made. At the completion of baseline data acquisition, the trial coordinator explains and asks the subject to take home two questionnaires (SF 36 and items from Active Australia) and a four-day diet diary for completion. The subject is supplied with a self-addressed express post envelope. Subjects are asked to complete and post these tasks within the following two weeks. Subjects are informed that visit two will be approximately six months after visit one. At the completion of visit one, the clinical trial coordinator will provide patients with a three months supply of trial medication as described in the section on randomization. Subjects will be instructed to take three tablets each day preferably with food. The same instructions are provided on the medication container. Finally, participants are provided with a pathology request form for collection of immunosuppressant levels post one-week initial consumption of trial medication (to assess if there is any change to the plasma immunosuppressive levels). Data obtained at the scheduled study visits and are transcribed onto case record forms for entry into a specifically designed Xanthin trial database. Subjects are contacted by telephone ten weeks after visit one, to ascertain their progress with trial medication adherence. The next three months of trial medications will be supplied to them by post. Subjects are then reminded that they will be contacted by telephone in two months time to confirm an appointment date and time for visit two.

**Table 2:** Outcome measures at baseline, 6 and 12 months

Primary Outcome Measures
Arterial stiffness – aortic pulse wave velocity (PWV)
Oxidative stress – plasma isoprostanes
Inflammation – plasma pentraxin 3
Secondary Outcome Measures
Arterial stiffness – augmentation index (AIx)
Arterial stiffness – carotid artery intimal medial thickness (CMIT)
Left ventricular overload – central pulse pressure
Vascular function – brachial artery reactivity (BAR)
Oxidative stress – plasma protein carbonyls
Antioxidant status – plasma total antioxidant capacity, - plasma antioxidant enzyme activities
- plasma antioxidant enzyme activities
Inflammation – C reactive protein, IL-6, IL-8, IL-10 and TNF-alpha
Additional blood measures – full blood profile, electrolytes, liver function
- lipid profile
- plasma immunosuppressant levels
- eGFR
Adverse events
Concomitant therapy
Self reported health – SF 36
Physical Activity – items from the Active Australia questionnaire
Nutrition – four day diet diary

### Visits two and three

These visits occur at six months and one year after baseline. An envelope containing a letter confirming appointment, pathology request forms, four-day diet diary, SF 36 and physical activity surveys will be mailed to patients. They will be asked to complete surveys and diet diary and to attend the pathology laboratory seven days prior to visits. Participants will also required to return empty tablet containers and containers with remaining tablets and issued with a further three-month supply of trial medication. At these visits, data acquisition from measures and tests described above will be performed again.

### Adherence to Study Medication

Sufficient medication is included in the containers to last for six months. However, as a crosscheck of the subjects adherence to drug therapy, each container has 300 tablets placed in it for each trial visit and the subjects are not informed that the containers have additional tablets. At each trial visit the subject is required to return the container. Two separate tablet counts are then conducted one by the trial coordinator and the trial pharmacist. This enables a check on adherence to therapy by calculating the difference between 300 and the days of therapy and comparing this with number of tablets left in the container.

### Withdrawal from Study

Subjects will be withdrawn from the study at their request, without prejudice, as documented and explained at the time of consenting. Patients who withdraw will be asked to consent to follow-up testing for the remainder of the trial to enable an intention to treat data analysis.

### Power calculation

Resource limitations will allow us to recruit 66 patients within a two-year period. Data from other studies indicates that Pentraxin 3 has the highest variability of the three outcome measures (mean ± SD; isoprostanes = 54.1 ± 7.0 [46], PWV = 11.4 ± 2.4 [47], pentraxin 3 = 0.75 ± 0.3 [48]). We assume that a decrease of 10% in pentraxin 3 would be clinically significant. Therefore, with alpha = 0.05, we will have about a 17% power to detect this 10% change in pentraxin 3 and a 52% power to detect a 20% change. This also allows for a 20% withdrawal rate. We recognize that the power of the study is small, but believe that the study will form the basis for progression to a larger study.

### Statistical analysis

All baseline continuous variables between groups will be compared using general linear modelling, whilst categorical variables will be compared using exact logistic regression. The rates of change will be estimated for each patient separately by linear regression. General linear modelling, will be used to compare the mean rate of change in primary outcome measures between groups, unadjusted and adjusted for actual and potential confounding variables. Mean differences, 95% confidence intervals and P-values will be corrected for repeated measures, and P-values corrected for multiple comparisons by the Holm method. All analyses will be performed using Stata/IC 10.1 for Windows (StataCorp LP, College Station, Tx).

## Discussion

The objective of the Xanthin trial is to assess whether the antioxidant astaxanthin can affect arterial stiffness, oxidative stress and inflammation in renal transplant patients. Data from this trial may lead to a larger scale intervention study assessing major cardiovascular endpoints. Although, previous studies using antioxidants have not generally resulted in cardiovascular benefits [19,20], we predict that because of its greater potency, astaxanthin will provide vascular benefits and reduce measures of oxidative stress and inflammation.

## Competing interests

The authors declare that they have no competing interests.

## Authors' contributions

RGF and JSC are responsible for the design of this clinical trial, the construction of the protocol and writing this manuscript. IKR provided statistical advice. DPG and HH provided technical advice and manuscript review. RD will perform measures of vascular structure and function and provided protocol design elements related to these. JES provided advice on vascular measures and manuscript review. All authors read and approved the final manuscript.

## Pre-publication history

The pre-publication history for this paper can be accessed here:



## Supplementary Material

Additional file 1 **Xanthin (ethics approvals).** Ethics approval for the trial. Click here for file

Additional file 2 **Xanthin funding letter.** An email from the Company that funded the trial ($13,000 USD). Click here for file
