# Supplementary material for: Astaxanthin vs placebo on arterial stiffness, oxidative stress and inflammation in renal transplant patients (Xanthin): a randomised controlled trial
Source: BMC Nephrol. 2008 Dec 18;9:17. doi: 10.1186/1471-2369-9-17 (PMC2666668; doi:10.1186/1471-2369-9-17)
Supplement: Additional file 1 — Xanthin (ethics approvals). Ethics approval for the trial. [file 1471-2369-9-17-S1.pdf]

Smith Marianne

From: Purse Lisa
Sent: Monday, 25 July 2005 8:48 AM
To: Smith Marianne
Subject: FW: Notification of Medical Ethics Approval: H0008414 Effects of antioxidant supplementation with Astaxanthin on blood vessels in renal transplant patients

-----Original Message-----
From: Rachael Cowen Kitanovic
[mailto:Rachael.Cowenkitanovic@utas.edu.au]
Sent: Friday, 22 July 2005 2:04 PM
To: Fassett Rob
Cc: Smith Maria; Purse Lisa; s4028781@student.uq.edu.au
Subject: Notification of Medical Ethics Approval: H0008414 Effects of antioxidant supplementation with Astaxanthin on blood vessels in renal transplant patients

[illegible]ear AssocProf Fassett,

Reference number: H0008414
Title: Effects of antioxidant supplementation with Astaxanthin on blood vessels in renal transplant patients

This study has been approved by the Northern Tasmania Health and Medical Human Research Ethics Committee.

A signed copy of the approval letter will be forwarded to you in the next few days. This will be sent to the first named investigator, except in the case of clinical trials where correspondence will be send to the named data manager, if applicable.

PLEASE NOTE REGARDING CLINICAL TRIALS: CTN and Indemnity Forms requiring signature will be forwarded to the respective signing authorities from our office with in 2 days. A signed copy of the trial approval documents will be forwarded once we have these documents back from the Ethics Committee Chair and the signing authorities at the relevant hospitals.

Should you have any queries please contact me on the below telephone [illegible]ber or this email address.

Kind regards,

--
Rachael Cowen Kitanovic
Health and Medical Ethics Officer
Research and Development Office
University of Tasmania
Private Bag 01
HOBART TAS 7001

Phone: (03) 62 26 1751
Fax: (03) 62 26 7148

http://www.research.utas.edu.au/human_ethics/index.htm

25.7.05 MI

Private Bag 01 Hobart
Tasmania 7001 Australia
Telephone (03) 6226 1751
Facsimile (03) 6226 7148
Rachael.Cowenkitanovic@utas.edu.au
http://www.research.utas.edu.au/

UTAS

MEMORANDUM

HUMAN RESEARCH ETHICS COMMITTEE (TASMANIAN) NETWORK

24 January 2006

Professor Rob Fassett
W.D. Booth Centre (Renal Research)
Level 5,
Launceston General Hospital
Charles Street
LAUNCESTON TAS 7250

Dear Professor Fassett

REF NO: **H8414**
TITLE: **Effects of antioxidant supplementation with Astaxanthin on blood vessels in renal transplant patients**

Application
Patient Information Sheet and Consent Form Version 2 dated 21/06/2005

The Northern Tasmania Health and Medical Human Research Ethics Committee considered and approved the above documentation at its meeting on **11th July 2005**.

All committees operating under the Human Research Ethics Committee (Tasmania) Network are registered and required to comply with the *National Statement on the Ethical Conduct in Research Involving Humans 1999* (NHMRC guidelines).

Therefore, the Chief Investigator's responsibility is to ensure that:

(1) The individual researcher's protocol complies with the HREC approved protocol.

(2) Modifications to the protocol do not proceed until **approval** is obtained in writing from the HREC.

(3) The confidentiality and anonymity of all research subjects is maintained at all times, except as required by law.

(4) Clause 2.37 of the *National Statement* states:

An HREC shall, as a condition of approval of each protocol, require that researchers immediately report anything which might warrant review of ethical approval of the protocol, including:

a. *Serious or unexpected adverse effects on participants;*

b. *Proposed changes in the protocol; and*

c. *Unforeseen events that might affect continued ethical acceptability of the project.*

30/1/06

The appropriate forms for reporting such events in relation to drug trials can be located at the website below. All adverse events must be reported regardless of whether or not the event, in your opinion, is a direct effect of the drug being tested.
http://www.research.utas.edu.au/rdo/ethics/human.htm

(5) All subjects must be provided with the current Patient Information Sheet and Consent Form as approved by the Ethics committee.

(6) The Committee is notified if any investigators are added to, or cease involvement with, the project.

(7) This study has approval for 4 years contingent upon annual review. A *Progress Report* is to be provided on the anniversary date of your approval. Your first report is due **11th July 2006**. You will be sent a courtesy reminder closer to this due date.

Clause 2.35 of the National Statement states:

As a minimum an HREC must require at regular periods, at least annually, reports from principal researchers on matters including:

a. *progress to date or outcome in the case of completed research;*
b. *maintenance and security of records;*
c. *compliance with the approved protocol; and*
d. *compliance with any conditions of approval*

(8) A *Final Report* and a copy of the published material, either in full or abstract, must be provided at the end of the project.

A copy of the signed CTN was received by fax and has been placed on our files. A copy of the HREC Membership list has been attached to this letter for your information.

Should you have any queries please do not hesitate to contact Rachael Cowen Kitanovic on (03) 62261751 in the first instance.

Yours sincerely

Amanda McAully, Executive Officer
HREC (TAS) Network

30.1.06

Private Bag 01 Hobart
Tasmania 7001 Australia
Telephone (03) 6226 1751
Facsimile (03) 6226 7148
Rachael.Cowenkitanovic@utas.edu.au
http://www.research.utas.edu.au/

UTAS

MEMORANDUM

HUMAN RESEARCH ETHICS COMMITTEE (TASMANIAN) NETWORK

23 May 2006

Associate Professor R Fassett
C/- Lisa Anderson / Marianne Smith
Renal Research Tasmania
W.D.Booth Centre
LAUNCESTON TAS 7250

Dear A/Prof Fassett

REF NO: **H0008414**
TITLE: **Effects of antioxidant supplementation with Astaxanthin on blood vessels in renal transplant patients**

Patient Information Sheet and Consent Form dated Version 3 dated 17th January 2006
Protocol Version 2.0 dated January 17 2006
Signed CTN for form Launceston General Hospital

The Northern Tasmania Health and Medical Tasmania Health and Medical Human Research Ethics Committee considered and approved the above documentation at its meeting on 20 March 2006.

All committees operating under the Human Research Ethics Committee (Tasmania) Network are registered and required to comply with the *National Statement on the Ethical Conduct in Research Involving Humans 1999* (NHMRC guidelines).

Should you have any queries please do not hesitate to contact Rachael Cowen Kitanovic on (03) 62261751 in the first instance.

Yours sincerely

RKitanovic

Rachael Cowen Kitanovic
Health and Medical Ethics Officer
On behalf of the Executive Officer
HREC (TAS) Network

Private Bag 01 Hobart
Tasmania 7001 Australia
Telephone (03) 6226 1751
Facsimile (03) 6226 7148
Rachael.Cowenkitanovic@utas.edu.au
http://www.research.utas.edu.au/

MEMORANDUM

UTAS

HUMAN RESEARCH ETHICS COMMITTEE (TASMANIAN) NETWORK

17 July 2006

A/Prof R Fassett
C/- Lisa Anderson / Marianne Smith
Renal Research Tasmania
W.D.Booth Centre
LAUNCESTON TAS 7250

Dear A/Prof Fassett

REF NO: **H0008414**
TITLE: **Effects of antioxidant supplementation with Astaxanthin on blood vessels in renal transplant patients**

Protocol Amendment Version 3.0 dated 31 March 2006
Patient Information Sheet and Consent Form Version 4 dated 31 March 2006

The Human Research Ethics Committee (Tasmania) Network considered and approved the above documentation at its meeting on 10/7/2006.

All committees operating under the Human Research Ethics Committee (Tasmania) Network are registered and required to comply with the *National Statement on the Ethical Conduct in Research Involving Humans 1999* (NHMRC guidelines).

Should you have any queries please do not hesitate to contact Rachael Cowen Kitanovic on (03) 62261751 in the first instance.

Yours sincerely

R Kitanovic

Rachael Cowen Kitanovic
Ethics Officer, Health and Medical
On behalf of the Executive Officer
HREC (TAS) Network
