# Supplementary material for: Astaxanthin vs placebo on arterial stiffness, oxidative stress and inflammation in renal transplant patients (Xanthin): a randomised controlled trial
Source: BMC Nephrol. 2008 Dec 18;9:17. doi: 10.1186/1471-2369-9-17 (PMC2666668; doi:10.1186/1471-2369-9-17)
Supplement: Additional file 2 — Xanthin funding letter. An email from the Company that funded the trial ($13,000 USD). [file 1471-2369-9-17-S2.doc]

To: Jeff Coombes, PhD                                                                            October 26, 2005
Senior Lecturer
School of Human Movement Studies
University of Queensland
 
Dear Dr. Coombes,
 
Thank you for your email and protocol for astaxanthin on oxidative stress.
I apologize for my delay in responding to you.
  
We, TKK and Cyanotech combined, would like to provide the amount of USD13,000.00 (Thirteen thousand US Dollars) for your study.
If you have any questions or comments, please let us know.
 
Sincerely yours. 
Nobuyuki Miyaji
Toyo Koso Kagaku Co., Ltd
